# An Account of Diseases in an Eastern District of London from June 20, to July 20, 1809

**Published:** 1809-08

**Authors:** 


### *An Account of Diseases in an Eastern District of London from June 20, to July 20, 1809.*

| | |
|---|---|
| ACUTE DISEASES. | |
| Typhus - - - - - - - | 2 |
| Scarlatina - - - - - - | 4 |
| Variola - - - - - - - - | 1 |
| Rheumatismus Acutus | 2 |
| CHRONIC DISEASES. | |
| Tussis - - - - - - - | 12 |
| Dyspnœa - - - - - - | 4 |
| Tussis cum Dyspnœa - | 8 |
| Phthisis Pulmonalis - | 4 |
| Hepatitis Chronica - . - | 3 |
| Hydrothorax - - - - - | 4 |
| Anasarca - - - - - - | 5 |
| Diarrhœa - - - - - | 3 |
| Menorrhagia Difficilis - | 2 |
| Obstipatio - - - - - - | 3 |
| Dysuria - - - - - - | 4 |
| Nephralgia - - - - - | 2 |
| Rheumatismus Chronicus | 7 |
| PUERPERAL DISEASES. | |
| Peritonitis - - - - - - | 1 |
| Abscessus Mammæ - - | 3 |
| Menorrhagia Lochialis | 5 |
| INFANTILE DISEASES. | |
| Pertussis - - - - - - | 7 |
| Convulsio - - - - - - | 2 |
| Febris Mesenterica - - | 2 |
| Aphthæ - - - - - - - | 4 |

The

The hooping cough has for some time been very prevalent, and in many instances very obstinate. This disease is of so frequent occurrence, and the symptoms of it are so well known, that there is no danger of its being mistaken for any other.

For these reasons it is generally managed by mothers or nurses, and but a small proportion of these cases come under regular medical treatment. By the interference of some friend, a fashionable or favorite remedy is introduced, and if the child recovers, every thing is attributed to this. It is, however, very certain, that in this, as well as in many other complaints, a varied mode of treatment is necessary in different temperaments, in different stages of the disease, and under the various circumstances which occur in the course of it. That this cannot be understood by persons in general is too evident to be denied or even questioned: it can only be known by those whose profession and habits lead them to take a comprehensive view of the subject. If there be any article of the materia medica which deserves the title of an infallible cure, or even of a specific remedy in the disease referred to, the probability is that such persons would be acquainted with it; but they know too much of the nature of the disease to pretend to any such discovery. They are aware, however, that in many cases, if their assistance were requested at an early period of the complaint, many of the symptoms might be mitigated, and much of the ill consequences attending them might be prevented.

